# P-1423. Effectiveness of nirsevimab against RSV-related hospitalizations in a pediatric primary care network using target trial emulation

**DOI:** 10.1093/ofid/ofaf695.1610

**Published:** 2026-01-11

**Authors:** Mahaa M Ahmed, Ziyi Wang, Torsten Joerger, Yun Li, Jeffrey S Gerber

**Affiliations:** Children's Hospital of Philadelphia, Philadelphia, PA; Children's Hospital of Philadelphia, Philadelphia, PA; Stanford University School of Medicine, Stanford, California; University of Pennsylvania, Philadelphia, Pennsylvania; Children's Hospital of Philadelphia, Philadelphia, PA

## Abstract

**Background:**

Respiratory syncytial virus (RSV) is a leading cause of lower respiratory tract infections and hospitalizations among infants. Nirsevimab is a monoclonal antibody to the RSV fusion protein that received U.S. FDA approval in July 2023 and recommended for all children < 8 months old. Real-world studies assessing the effectiveness of nirsevimab are needed.

**Figure:**
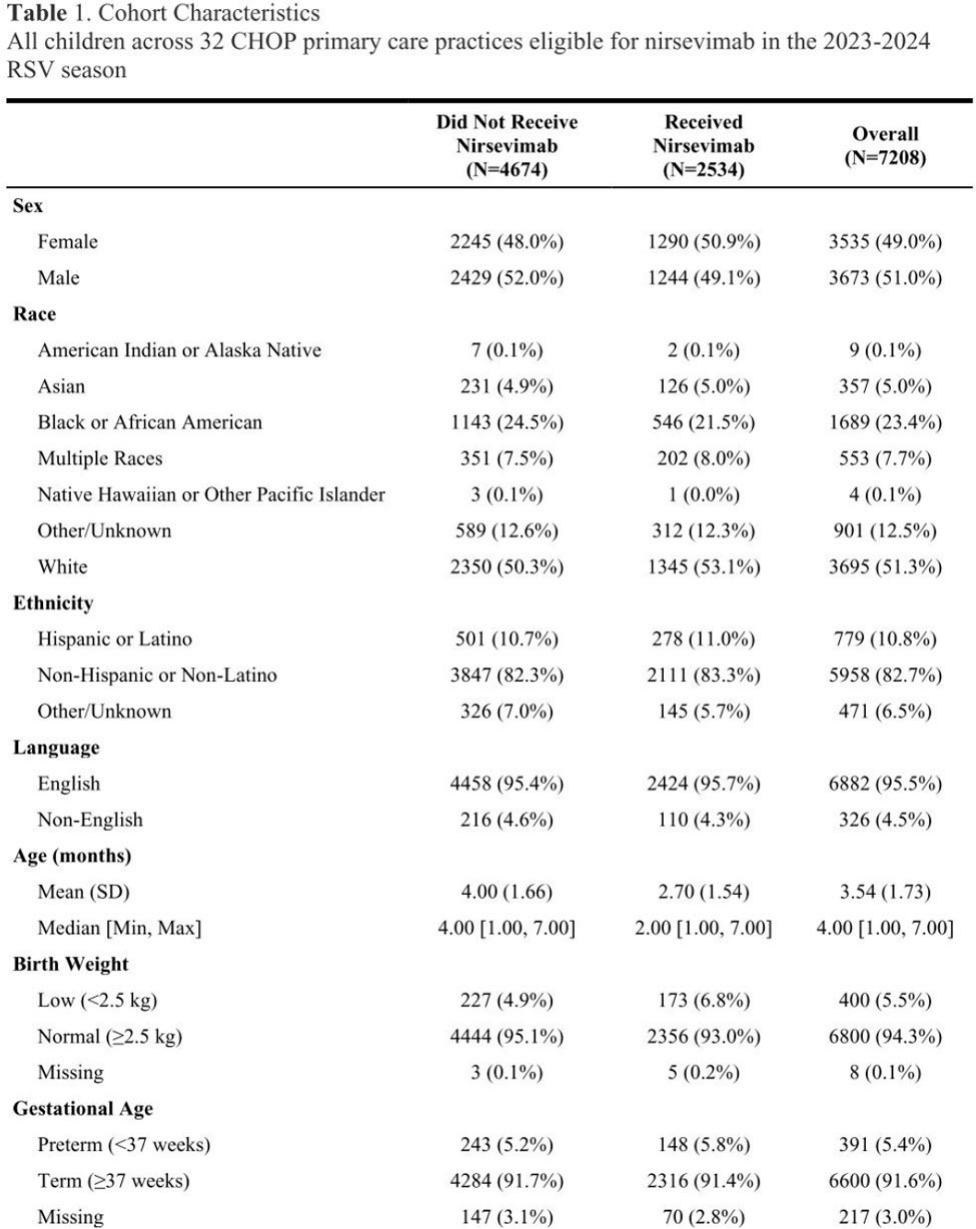

**Table 2.**
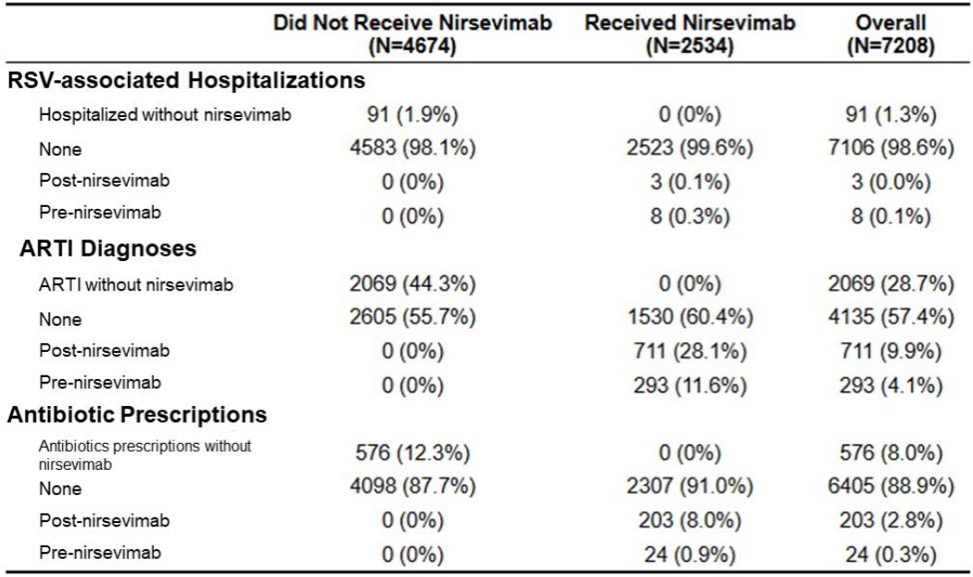
RSV hospitalizations, ARTI diagnoses, and antibiotic prescriptions among eligible patients

**Methods:**

Using target trial emulation, we assessed the effectiveness of nirsevimab across a diverse pediatric primary care network over the 2023-2024 RSV season (October 1 to March 31). To establish a primary care attendee cohort of infants eligible for nirsevimab who remained in the network, we included patients < 8 months old on October 1 with at least one primary care visit within 14 days of birth and at least one primary care visit after turning 8 months old or after the end of RSV season. The primary outcome was an encounter with both a positive RSV laboratory test and hospitalization with an ICD-10 code for RSV bronchiolitis/pneumonia. Secondary outcomes included acute respiratory tract infections (ARTIs) and antibiotic prescriptions for ARTIs during RSV season. Sequential target trial emulation was used and adjusted for follow-up time in monthly intervals for a total of five months.

**Figure 1.**
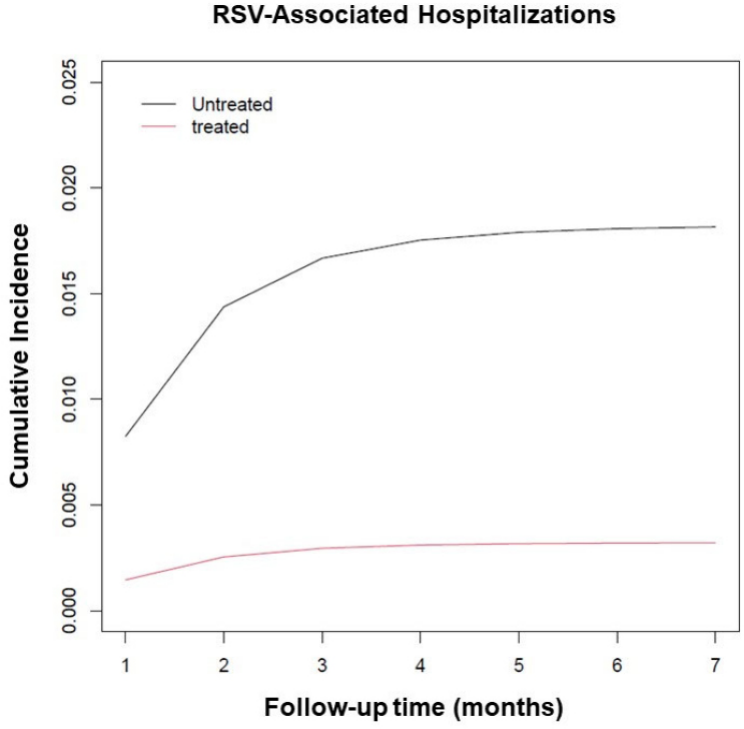
Cumulative incidence of RSV-associated hospitalizations among treated vs. untreated patients

**Figure 2.**
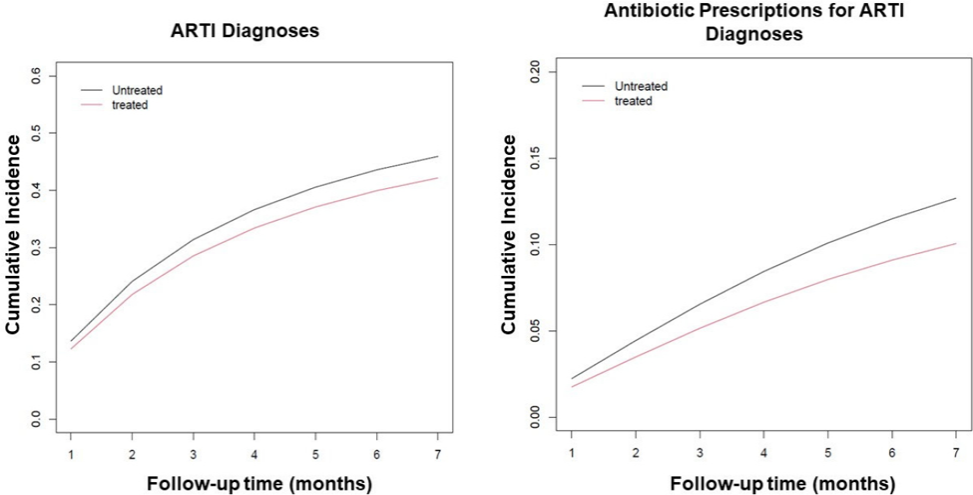
Cumulative incidence of ARTI diagnoses and antibiotic prescriptions among treated vs. untreated patients

**Results:**

Our study cohort includes 7,208 patients. Among them, 35% of eligible patients received nirsevimab. There were 91 of 4,674 (1.9%) RSV-associated hospitalizations among patients who did not receive nirsevimab and 3 of 2,534 (0.1%) RSV-associated hospitalizations among patients who received nirsevimab. Overall, trial emulation demonstrated that patients who received nirsevimab had a significantly lower rate of RSV-associated hospitalization than those who did not given that the 5-month risk difference for RSV-associated hospitalizations was -0.015 (95% CI: -0.019, -0.001). Children receiving nirsevimab also had fewer ARTI encounters with a 5-month risk difference of -0.038 (95% CI: -0.060, -0.015) and fewer antibiotic prescriptions with a 5-month risk difference of -0.026 (95% CI: -0.040, -0.012).

**Conclusion:**

Nirsevimab was highly protective against RSV-associated hospitalizations among infants entering their first RSV season and reduced both ARTI encounters and antibiotic use.

**Disclosures:**

All Authors: No reported disclosures

